# Urban mobility resilience and spatial–temporal equity under public health crises: longitudinal evidence from China’s ride-hailing sector (2019–2024)

**DOI:** 10.3389/fpubh.2026.1831678

**Published:** 2026-08-06

**Authors:** Baiying Sun, Zhengfeng Huang, Kehong Guan, Pengjun Zheng

**Affiliations:** Faculty of Maritime and Transportation, Ningbo University, Ningbo, China

**Keywords:** longitudinal analysis, public health emergencies, shared urban mobility, social equity, socio-ecological resilience, spatial–temporal disparities

## Abstract

**Introduction:**

Shared urban mobility systems are critical infrastructure for ensuring essential travel and maintaining socioeconomic stability during public health crises. However, the long-term system resilience and spatial–temporal equity of these networks under severe macro-shocks (e.g., the COVID-19 pandemic) and subsequent recovery phases remain underexplored.

**Methods:**

Utilizing a comprehensive longitudinal dataset (2019–2024) from Ningbo, China, this study constructs a multi-dimensional evaluation framework encompassing market supply, operational performance, economic viability, and service equity. We applied a projection pursuit-weighted TOPSIS model to quantify the overall resilience trajectory. Furthermore, to uncover structural disparities in service access, tensor decomposition and the Theil index were employed to measure the spatial–temporal distribution of passenger waiting times.

**Results:**

The empirical findings reveal that the socio-ecological resilience of the ride-hailing system exhibited severe vulnerability during the initial COVID-19 outbreak (Q1 2020), with closeness values dropping to 0.290. While overall resilience peaked during the post-pandemic recovery (Q4 2023), the system experienced a structural “involution,” characterized by continuous supply expansion that outpaced demand, leading to degraded per-vehicle operational efficiency. Moreover, the study identified significant spatial–temporal inequalities in mobility access during pandemic restrictions (2020–2021), which were progressively mitigated following strict regulatory interventions and market normalization.

**Conclusion:**

This six-year longitudinal analysis highlights the dynamic vulnerability and adaptive capacity of urban mobility networks under prolonged public health emergencies. To build crisis-resilient cities, policymakers must implement data-driven, dynamic supply–demand regulatory mechanisms to prevent resource misallocation, safeguard the livelihoods of gig workers, and ensure equitable access to essential mobility services across all urban sectors.

## 1 Introduction

The digital and sharing economy has rapidly reshaped urban mobility patterns, transforming ride-hailing networks from mere commercial services into essential infrastructure for urban socio-ecological resilience (1). Under normal circumstances, these platforms dynamically match supply and demand to optimize resource allocation (2, 3). However, during extreme external disturbances—such as the COVID-19 pandemic—ride-hailing systems serve as a critical lifeline, ensuring essential mobility access for the public and maintaining basic socioeconomic operations (4). Consequently, the system resilience of the ride-hailing industry is intrinsically linked to a city’s overarching capacity to withstand, adapt to, and recover from prolonged public health crises and macro-environmental shocks (5, 6).

Despite a continuous expansion in market scale and supply redundancy in recent years, the ride-hailing system has exhibited significant sensitivity and vulnerability when facing severe shocks (5, 7). The recurring waves of the pandemic and subsequent economic fluctuations have severely tested the industry’s operational mechanisms (8). On one hand, the imbalance between stagnant demand and blind supply expansion has triggered a structural “involution” (excessive internal competition), severely deteriorating per-vehicle operational efficiency and the economic viability of gig workers (9). On the other hand, driven by profit-seeking behaviors during supply shortages, platforms often adopt biased dispatching strategies that exacerbate the unequal allocation of service resources (10, 11). This inequity directly undermines spatial–temporal mobility justice, disproportionately affecting vulnerable populations and testing the ultimate service quality and equity resilience of the urban transport network (12).

While an emerging body of literature has begun to explore the ride-hailing industry through dimensions such as market supply and operational status (13, 14), existing research has also provided an important basis for resilience indicator selection. In general, market supply resilience is commonly measured by indicators such as the total number of ride-hailing vehicles (15, 16), the number of online operational vehicles (17), and the number of platforms (18) or leasing companies, which are fundamental components in defining the economic supply curve of ride-hailing systems (17). Operational status resilience is often reflected by the number of completed orders (18), average daily working hours (16), average daily operating mileage (16), average passenger-carrying time per vehicle (19), and average monthly net profit (19). Cost and profitability resilience is usually assessed using indicators related to operating costs (15, 20), operating income, and driver profits (21). Service quality and equity resilience mainly involves passenger waiting time (22, 23), driver dispatch equity (24, 25) and driver income equity. However, two critical gaps remain in understanding its resilience under prolonged crises. First, existing studies evaluating service equity predominantly rely on static administrative zoning or overarching statistical distributions (e.g., standard deviation, Gini coefficient) (26, 27). These approaches obscure the dynamic spatial–temporal heterogeneity of mobility pressure (e.g., disparities between urban cores and peripheries across different times of day), failing to identify the underlying “structural sources” that undermine equity resilience (28, 29). Second, the majority of research has been confined to short-term impact assessments or specific cross-sectional time points (7, 30). There is a pronounced lack of longitudinal tracking that captures the evolutionary trajectory of system resilience across different developmental stages—from pre-crisis stability through pandemic disruptions to post-crisis recovery and regulatory adjustments (6).

To address these critical gaps, this study establishes a comprehensive multi-dimensional framework to evaluate the long-term system resilience evolution of the ride-hailing industry, utilizing a robust quarterly dataset (2019–2024) from Ningbo, China. We construct the evaluation system across four core dimensions: market supply, operational status, cost and profitability, and service quality and equity resilience. Methodologically, we introduce a tensor decomposition approach to automatically identify dynamic spatiotemporal mobility patterns without relying on predefined geographic zones, which is then coupled with the Theil index to profoundly dissect the structural inequities in passenger service access (31). By employing a projection pursuit-weighted TOPSIS model to quantify the resilience levels (32, 33), this six-year longitudinal study provides deep insights into the internal dynamics of shared mobility networks under external disturbances. The findings offer vital empirical evidence for government regulators and public health policymakers to optimize resilience-oriented interventions, enhance disturbance response capabilities, and safeguard equitable resource allocation in urban transport systems (34).

## 2 Methods

### 2.1 Construction of the socio-ecological resilience framework

We conceptualize and deconstruct the urban ride-hailing network within the classical Socio-Ecological Systems (SES) theoretical framework established by Holling (1973) (35) and Ostrom (2009) (36). The ride-hailing network is far from being merely a commercial passenger transport platform or a technological intermediary; rather, it is a typical, highly coupled Urban Transportation Socio-Ecological System (UT-SES). Within this system, the urban road network, physical vehicles, and the spatiotemporal distribution of transport capacity collectively constitute the Resource System and Resource Units of the complex network. Meanwhile, drivers, passengers, platform algorithms, and regulatory agencies intertwine to form a dynamically interacting set of Actors and Governance Systems. Drawing upon socio-ecological resilience theory and long-term industry monitoring, the framework encompasses four core dimensions with 19 quantitative sub-indicators:

(1) Market Supply Resilience: Reflects the structural foundation and supply redundancy necessary to maintain essential urban mobility during crises (e.g., number of operational vehicles, platforms). It reflects the system’s physical redundancy and resource mobilization capacity during the initial phase of a shock, characterizing the buffer capacity of the physical subsystem.
(2) Operational Status Resilience: Measures the system’s ability to maintain efficient service delivery and avoid internal resource waste under fluctuating pandemic restrictions (e.g., daily served orders, utilization rates). It portrays the supply–demand matching efficacy and self-organization efficiency under dynamic restrictions, demonstrating the system’s capacity for self-recovery.
(3) Cost and Profitability Resilience: Evaluates the economic viability and survival capacity of gig workers, which directly dictates the long-term stability of the labor pool (e.g., average net profit, leasing fees). It directly characterizes the livelihood adaptability and vulnerability of the core actors within the social subsystem (i.e., gig workers), which serves as the endogenous economic foundation for maintaining the stability of the labor pool.
(4) Service Quality and Equity Resilience: Assesses the spatial–temporal justice of resource allocation, ensuring that vulnerable populations and peripheral urban areas maintain access to basic mobility services. It ultimately maps to the spatial–temporal justice and inclusive governance outcomes, measuring whether resource deprivation or spatial exclusion occurs under extreme systemic pressure.

The longitudinal evolutionary trajectories of these four dimensions comprehensively replicate the dynamic adaptive behaviors of a complex system throughout its entire lifecycle of shock—absorption—self-organization—reconstruction. Consequently, the analytical perspective of this framework has thoroughly shifted from the industry’s conventional static benefit-output evaluation to exploring the survival, adaptation, and evolutionary mechanisms of a complex system under uncertain shocks.

Table 1 presents all secondary indicators of resilience evaluation index system.

**Table 1:** Resilience evaluation index system.

Indicator	Sub-indicator	Direction
Market supply resilience	Number of registered ride-hailing vehicles	+
	Number of online operational vehicles	+
	Vehicle utilization	+
	Number of ride-hailing leasing companies	+
	Number of ride-hailing platforms	+
Operational status resilience	Total served orders	+
	Daily served orders per vehicle	+
	Average daily passenger-carrying distance per vehicle	+
	Average daily revenue per vehicle	+
	Average daily passenger-carrying time per vehicle	+
	Average daily working hours per vehicle	+
Cost and profitability resilience	Average monthly net profit per vehicle	+
	Average monthly insurance fee per vehicle	−
	Fuel cost per kilometer	−
	Average monthly vehicle leasing fees	−
Service quality and equity resilience	Number of driver complaints received by platforms	−
	Average passenger waiting time	−
	Driver dispatch equity	−
	Passenger waiting time equity	−

### 2.2 Quantifying spatial–temporal equity in mobility access

A resilient urban transport network must ensure equity for both service providers (drivers) and end-users (passengers). We introduce the Gini coefficient, Tensor Decomposition, and Theil index to map the structural disparities exacerbated by public health emergencies.

#### 2.2.1 Driver dispatch equity (Gini coefficient)

Driver-side equity reflects the platform’s ability to maintain fair income distribution and safeguard gig workers’ livelihoods during market contractions. We quantify this using the Gini coefficient based on the average revenue per order (37). Drivers are ranked and divided into n groups. Let *P_i_* denote the cumulative proportion of drivers and *W_i_* denote their cumulative proportion of total revenue. The Gini coefficient is shown as Equations 1–3:

$$\text{Gini}=1-\sum_{i=1}^{n}\;(O_{i}+O_{i-1})\cdot (D_{i}-D_{i-1})$$ (1)

$$D_{i}=\sum_{k=1}^{i}\;P_{k}$$ (2)

$$O_{i}=\sum_{k=1}^{i}\;W_{k}$$ (3)

A lower value indicates stronger equity resilience in safeguarding driver welfare.

#### 2.2.2 Passenger waiting time equity (tensor decomposition and Theil index)

Unlike traditional studies that rely on static administrative zones, this study introduces a data-driven tensor decomposition approach to identify dynamic spatiotemporal mobility clusters without predefined geographic borders (38, 39). This is crucial for capturing sudden shifts in travel behavior during pandemic lockdowns.

We construct a third-order tensor $T\in R^{I\times J\times K}$ representing the origin, destination, and hourly time intervals of ride-hailing trips. Using CANDECOMP/PARAFAC (CP) decomposition, the tensor is approximated by R latent spatiotemporal patterns is shown as Equation 4:

$$T\approx \hat{T}=\sum_{r=1}^{R}\;a_{r}\circ b_{r}\circ c_{r}$$ (4)

where vectors *a_r_*, *b_r_*, *c_r_* represent the spatial generation, spatial attraction, and temporal intensity of the *r*-th mobility pattern, respectively. The optimal rank *R* is determined by minimizing the Root Mean Square Error (RMSE) (40).

Based on these identified patterns, we apply the Theil index to evaluate the inequality of average passenger waiting times (41). The Theil index (*T*) is uniquely suited for this as it decomposes overall inequality into between-group (*T_B_*, temporal disparity) and within-group (*T_W_*, spatial disparity) components is shown as Equations 5, 6:

$$T=\sum_{k=1}^{K}\;\sum_{g=1}^{n_{k}}\;(\frac{Y_{\mathrm{kg}}}{Y})\ln(\frac{Y_{\mathrm{kg}}/Y}{P_{\mathrm{kg}}/P})$$ (5)

$$T=T_{W}+T_{B}$$ (6)

Here, *Y_kg_* and *P_kg_* denote the total waiting time and order volume for spatial sub-tensor g in time group *k*, respectively. This decomposition enables precise diagnosis of whether service inequity stems from temporal demand shocks or persistent spatial discrimination.

### 2.3 System resilience quantification via PP-TOPSIS

To avoid subjective bias in weighting and to handle the continuous upward trend of certain variables over the six-year observation period (32), we integrate a Projection Pursuit (PP) model with the Technique for Order Preference by Similarity to Ideal Solution (TOPSIS).

First, the original indicator matrix $X={\left[R_{\mathrm{ij}}\right]}_{\mathrm{nq}}$ ( n and q denote the number of evaluation objects and indicators, respectively) is transformed via Min-Max normalization into *r_ij_* ∈[0,1] to eliminate dimensional differences (33). Next, the PP model projects the high-dimensional data into a one-dimensional value $Z_{i}=\sum_{j=1}^{q}\;a_{j}r_{\mathrm{ij}}$ . The optimal projection direction *a_j_* is determined by maximizing the objective function *Q*(*a*), as expressed in Equation 7:

$$\max Q(a)=S_{z}\cdot D_{z}$$ (7)

where *S_z_* and *D_z_* represent the global standard deviation and local density of the projection values, respectively. The optimization is subject to the following constraint is shown as Equation 8:

$$s.t.\sum_{j=1}^{q}\;a_{j}^{2}=1;-1<a_{j}<1$$ (8)

A Particle Swarm Optimization (PSO) algorithm is deployed to solve this nonlinear optimization, yielding the objective indicator weights $w_{j}=a_{j}^{2}$ .

Finally, the TOPSIS method is utilized to compute the relative closeness degree (*C_i_*) of the mobility system’s resilience for each quarter, and its shown as Equation 9. Based on the weighted normalized matrix, the Euclidean distances to the positive ideal solution ( $D_{i}^{+}$ ) and negative ideal solution ( $D_{i}^{-}$ ) are calculated:

$$C_{i}=\frac{D_{i}^{-}}{D_{i}^{+}+D_{i}^{-}}$$ (9)

The value of *C_i_* ranges from 0 to 1, providing a standardized, objective measure of the system’s capacity to absorb shocks and sustain essential services during varying phases of the public health crisis.

## 3 Data preparation

**Figure 1:**
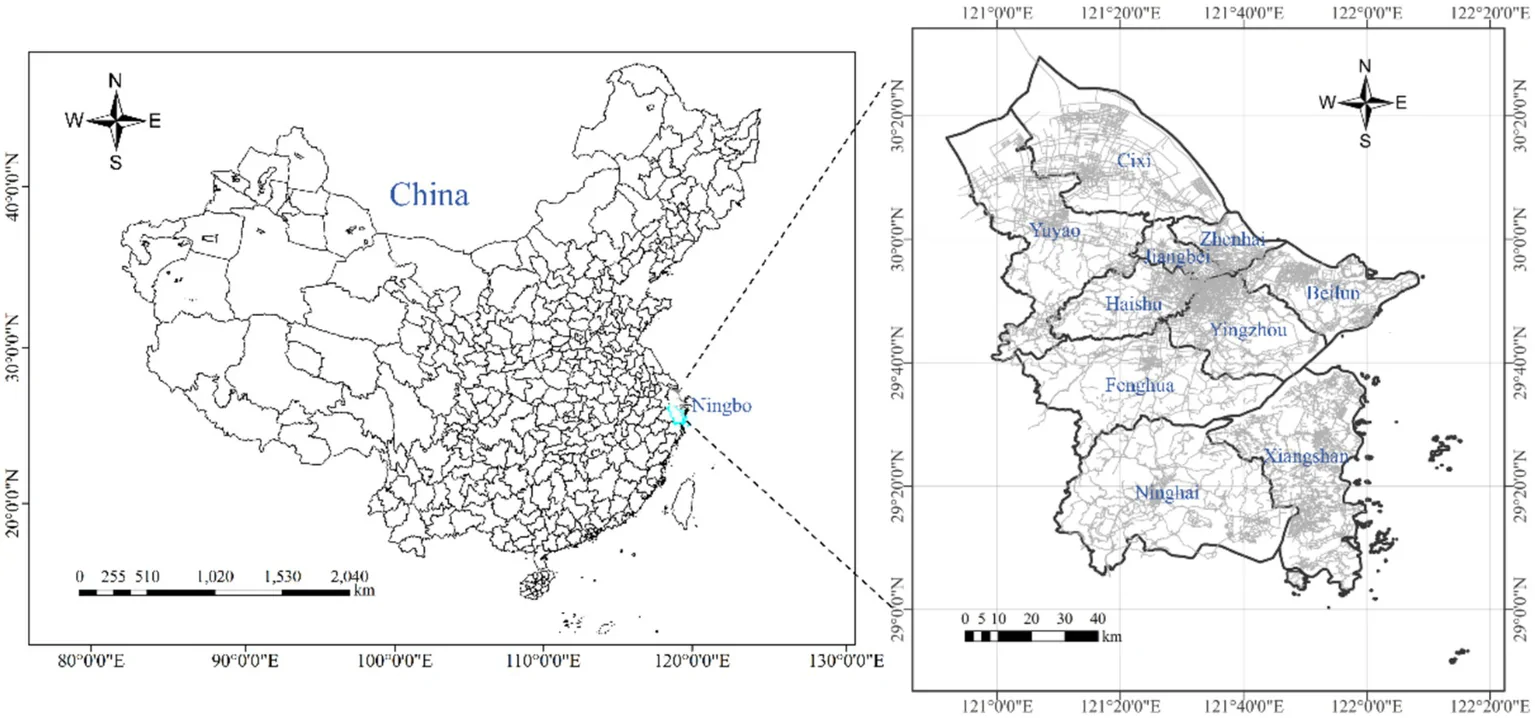
Geographical location and administrative divisions of Ningbo City.

This study selects the ride-hailing industry in Ningbo, China, as a case for evaluating system resilience performance. Ningbo is a coastal city located in eastern Zhejiang Province, situated on the southern wing of the Yangtze River Delta, bordering Shanghai to the north. The total land area of Ningbo is 9,365.58 square kilometers. The administrative divisions of Ningbo are shown in Figure 1. As of the end of 2024, Ningbo had a resident population of 9.777 million, including 1.889 million in Cixi City, 1.711 million in Yinzhou District, 1.267 million in Yuyao City, 1.072 million in Haishu District, 904,000 in Beilun District, and 2.934 million across other regions. The authors have provided long-term monitoring services for the taxi and ride-hailing industries in Ningbo. The dataset used in this study includes quarterly records from 2019 to 2024, covering vehicle information, driver profiles, ride-hailing operation data, and driver complaints against platforms. Specifically, the ride-hailing operation data contain detailed attributes such as vehicle plate ID, trip date, travel distance, fare revenue, pickup and drop-off coordinates, and pickup/drop-off times. The data sources are as follows: ride-hailing operation data were obtained from the Ride-hailing Regulatory Division of the Ningbo Municipal Transportation Bureau; platforms, ride-hailing leasing companies, vehicles information were obtained from the Ningbo Highway and Transport Management Center; cost and profitability data were obtained from the Ningbo Ride-hailing Association and local ride-hailing leasing companies.

**Figure 2:**
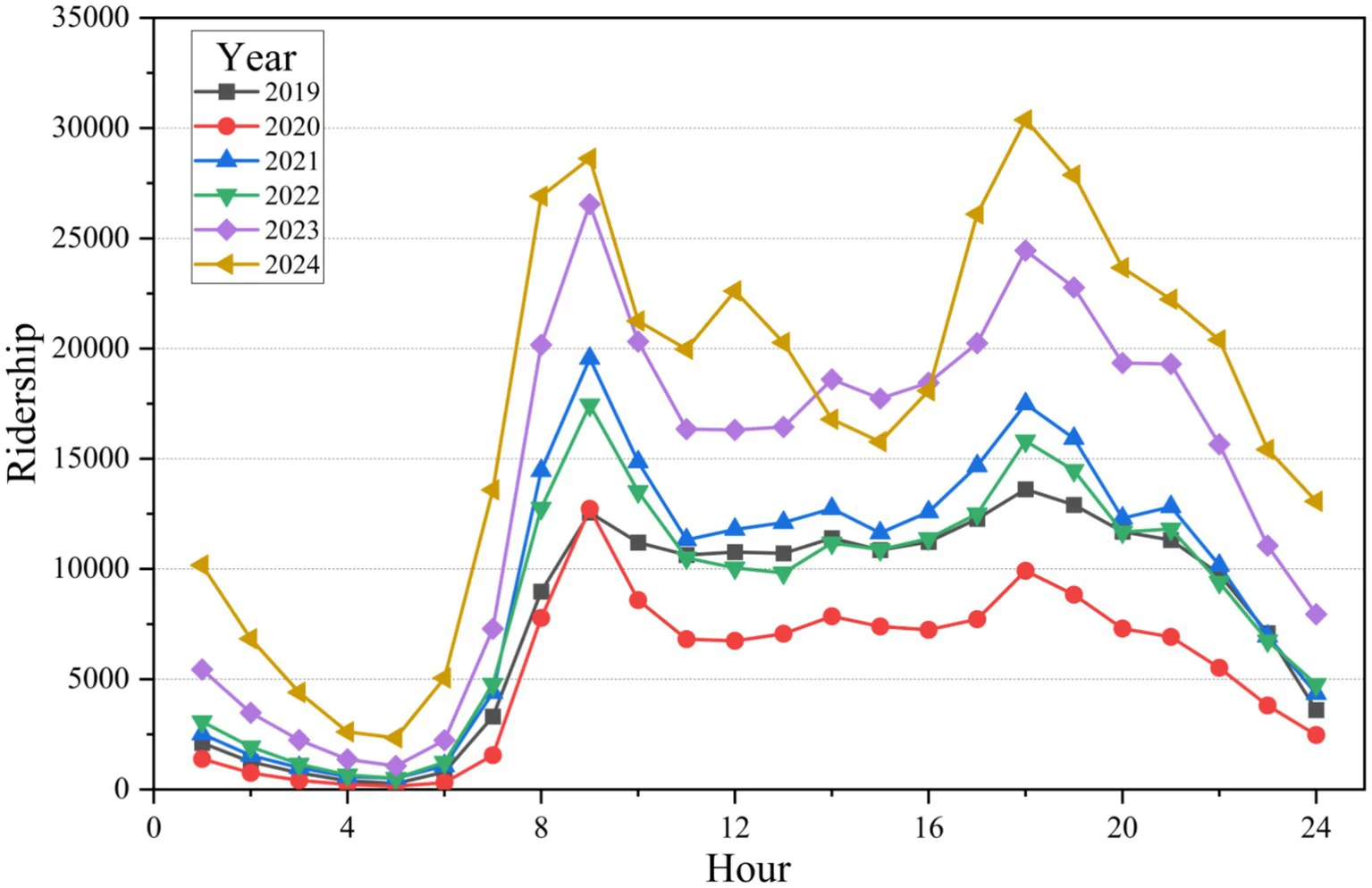
Hourly variation in ridership.

We processed the ride-hailing orders as follows to prepare for the analysis of driver dispatch equity. First, we calculated the average daily number of orders on weekdays from 2019 to 2024. Then, the daily data were disaggregated by hour to depict the hourly variation patterns in order volume, as illustrated in Figure 2. The temporal trends in passenger flow reveal a typical diurnal peak-valley pattern, with clear morning and evening rush hours. Specifically, peak periods are observed from 7:00 to 9:00 (morning peak) and 17:00 to 19:00 (evening peak), while off-peak periods span from 9:00 to 17:00. The data show that passenger demand increases significantly during both peak periods. From a year-over-year perspective, order volumes in 2023–2024 show a marked increase. This is likely a consequence of economic recovery; as the ride-hailing industry has relatively low entry barriers, it has served as a labor absorption buffer, attracting a growing number of job seekers. In contrast, order volumes in 2020 were significantly lower than in other years, primarily due to the impact of the COVID-19 pandemic, which caused a sharp decline in travel demand and further tested the industry’s operational resilience.

## 4 Results

### 4.1 Spatiotemporal mobility disparities under pandemic shocks

**Figure 3:**
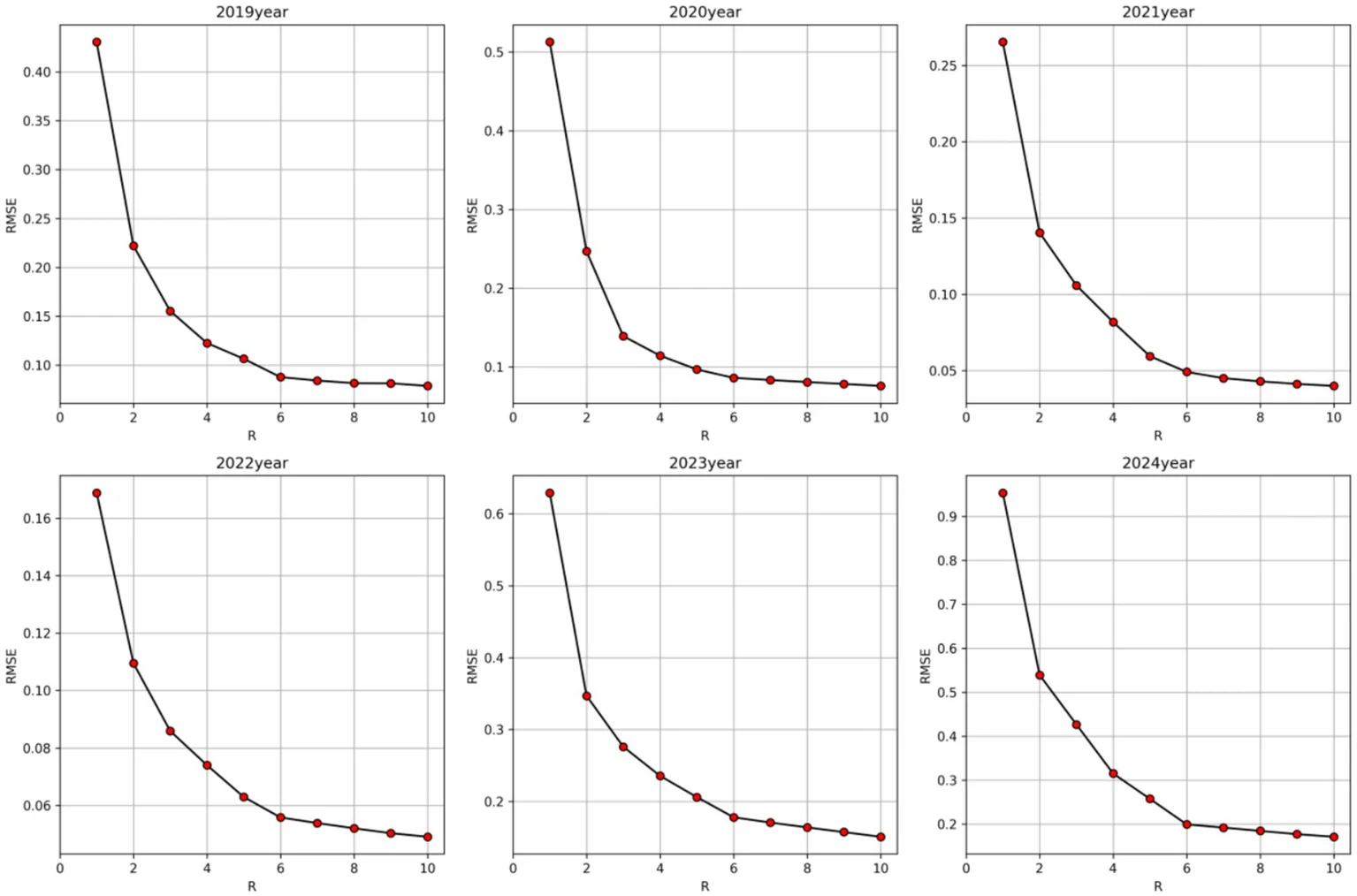
RMSE under different ranks.

As to the spatiotemporal grouping, The RMSE of tensor reconstruction from 2019 to 2024 under varying decomposition ranks R is shown in Figure 3. As the value of R increases, the decomposition error gradually decreases. When R is small, the RMSE drops significantly with each increment. However, once R approaches 6, the rate of error reduction becomes notably slower. Therefore, to ensure both accuracy and comparability across quarters, this study sets the decomposition rank to R=6 .

**Figure 4:**
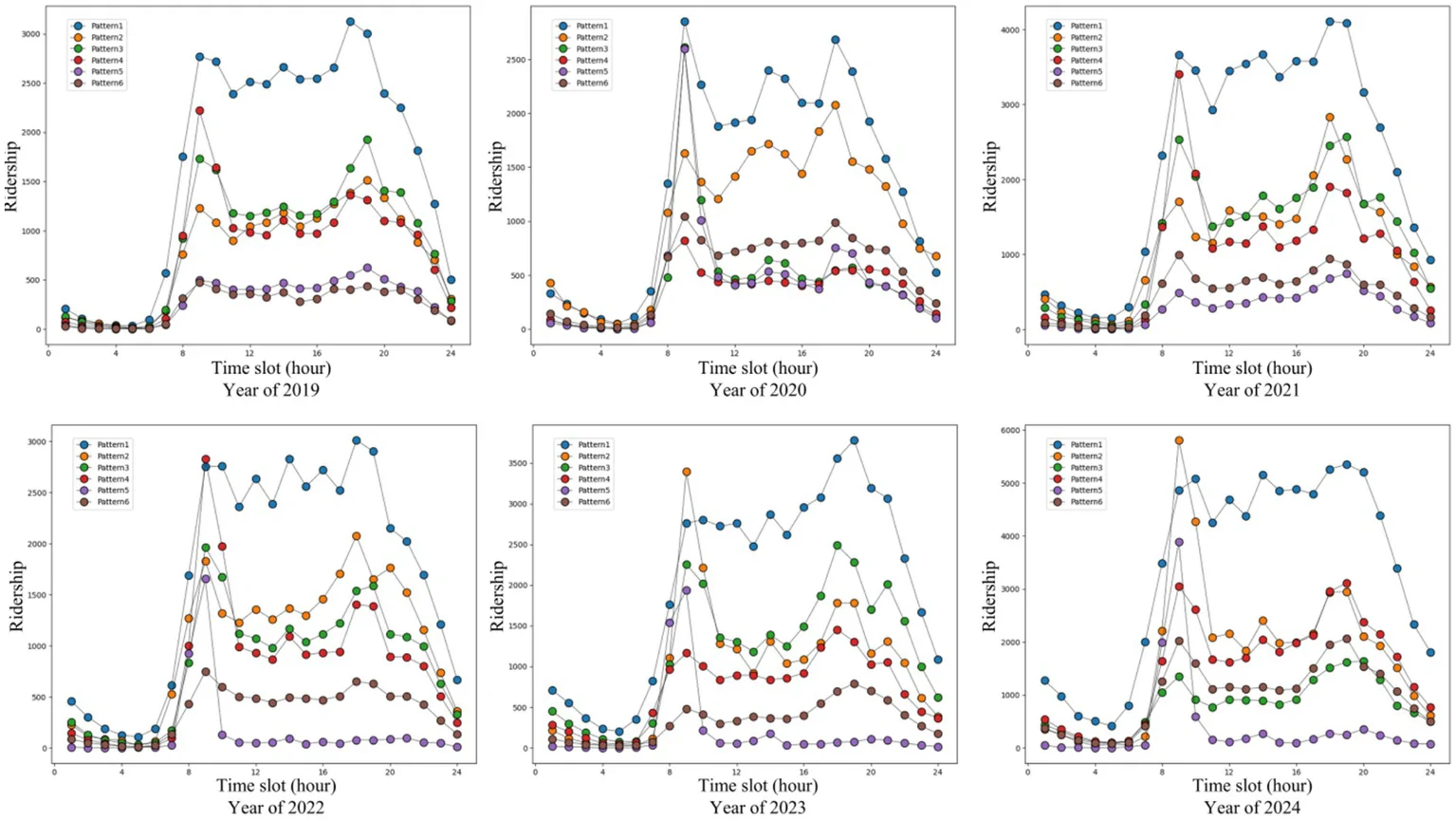
Temporal variation in ridership across decomposed patterns.

The hourly passenger pick-up volume changes for the 6 patterns extracted from the daily average order data from 2019 to 2024 are shown in Figure 4. The temporal trends of these patterns closely resemble those observed in Figure 2, and can be clustered into three representative time-based categories: morning peak (7:00–9:00), off-peak (9:00–17:00), and evening peak (17:00–19:00). These temporal groupings serve as the basis for the “between-group” dimension in the subsequent Theil Index–based equity analysis.

**Figure 5:**
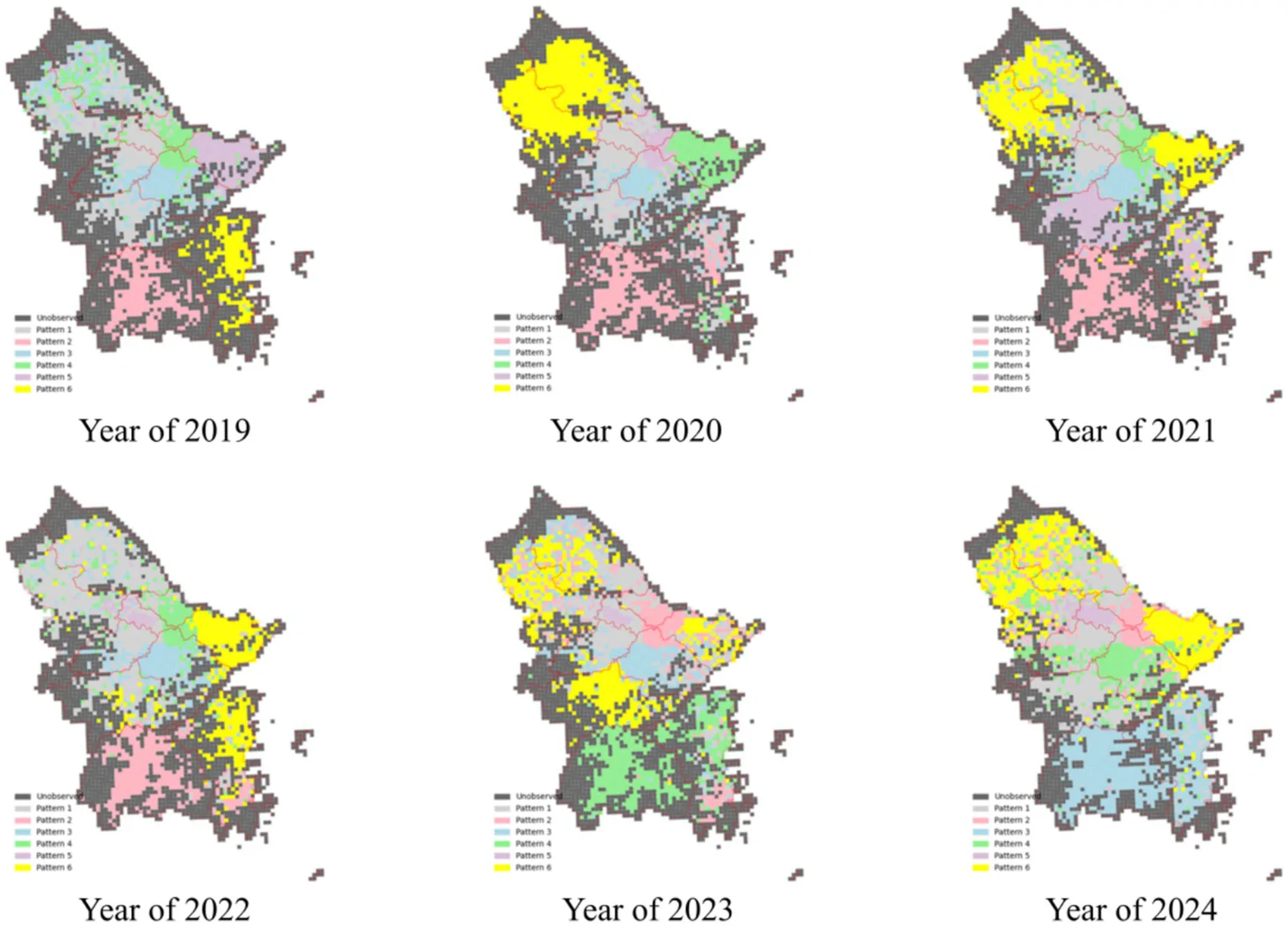
Urban spatial zoning based on travel pattern decomposition.

To further illustrate the spatial structure of mobility patterns, Figure 5 presents the spatial distribution of travel modes based on origin features extracted through tensor decomposition. Different colors in Figure 5 represent distinct travel patterns, helping to reveal the dominant spatial structures of intra-urban travel behavior and providing a basis for subsequent within-group comparative analysis. The matching between the patterns and grid cells is based on the origin factor matrix obtained from tensor decomposition. The factor value for each grid cell shows how much its pick-up activity aligns with each spatiotemporal pattern. We select the mode with the highest factor value as the representative pattern for that grid cell. This means we assign the cell to the pattern where it has the highest passenger pick-up volume.

According to the clustering results of ride-hailing passenger pick-up patterns in Figures 4, 5, the majority of spatiotemporal patterns exhibit relatively stable temporal dynamics and spatial aggregation characteristics. However, some patterns show notable variations in certain years, for example, in 2020, a period when the industry’s operational and equity resilience was severely tested by the COVID-19 pandemic.

Pattern 1 in Figure 4 is the dominant mode with the highest passenger pick-up volume. Its spatial configuration clearly reflects Ningbo’s core urban structure. This pattern primarily centers around Haishu District which is the traditional city center. This pattern also consistently integrates with high-demand surrounding counties such as Cixi and Yuyao. Temporally, Pattern 1 is characterized by persistently high demand from 9:00 a.m. to 9:00 p.m., with a particularly prominent evening peak. This aligns with the typical travel behavior of high-density, multifunctional urban cores, where both daytime commuting and evening leisure trips are prevalent.

In contrast, 2020 witnessed significant deviations. In that year, Yuyao and Cixi were primarily associated with Pattern 6, which exhibited a temporally independent profile with consistently low demand for travel throughout most time of the day. Although a modest morning peak remained, there was almost no evening peak, and nighttime travel was also rare. This may be due to residents reducing unnecessary outings during the pandemic, leading to a decline in ride-hailing demand during the latter half of the day, and further reflecting the vulnerability of travel demand patterns in county areas under the impact of public health emergencies.

Building upon the identified spatiotemporal patterns, this study uses the Theil Index to quantitatively assess the equity of passenger waiting times. The advantage of the Theil Index lies in its decomposability, which allows the overall inequality (Theil Index) to be split into between-group disparity (service imbalance across different time periods) and within-group disparity (service gaps among different spatial pattern groups). The quarterly Theil Index results for passenger waiting time equity from 2019 to 2024, along with the contribution rates of between-group and within-group disparities, are presented in Table 2.

**Table 2:** Decomposition of the Theil index in different quarters

Quarter	Theil Index	Between-group contribution rate	Within-group contribution rate	Quarter	Theil Index	Between-group contribution rate	Within-group contribution rate
2019Q1	0.0184	28.60%	71.40%	2022Q1	0.0053	47.20%	52.80%
2019Q2	0.0098	35.50%	64.50%	2022Q2	0.0075	21.20%	78.80%
2019Q3	0.0042	21.40%	78.60%	2022Q3	0.0043	26.20%	73.80%
2019Q4	0.0097	37.40%	62.60%	2022Q4	0.0041	29.10%	70.90%
2020Q1	0.0058	13.70%	86.30%	2023Q1	0.0069	53.00%	47.00%
2020Q2	0.0105	31.50%	68.50%	2023Q2	0.0069	21.80%	78.20%
2020Q3	0.0186	34.00%	66.00%	2023Q3	0.0043	6.00%	94.00%
2020Q4	0.0211	24.10%	75.90%	2023Q4	0.0037	7.50%	92.50%
2021Q1	0.0216	34.90%	65.10%	2024Q1	0.0054	34.50%	65.50%
2021Q2	0.0199	52.70%	47.30%	2024Q2	0.0027	14.30%	85.70%
2021Q3	0.0072	29.80%	70.20%	2024Q3	0.0013	49.60%	50.40%
2021Q4	0.0079	89.10%	10.90%	2024Q4	0.0033	40.70%	59.30%

Analysis of the results in Table 2 shows that the evolution of passenger waiting time equity can be described by two distinct phases. The first phase lasted from 2019 to Q2 2021. Compared to the subsequent period, the overall equality during this phase was at a relatively low level (corresponding to high Theil Index values) and showed significant volatility. From Q3 2021 to the end of 2024, with the strengthening of government regulation, the overall equity of passenger waiting times improved significantly: the Theil Index showed a fluctuating downward trend, decreasing from 0.0072 to 0.0033 and reaching a historical low of 0.0013 in Q3 2024.

From the perspective of group contribution decomposition, the between-group contribution rate is relatively high in Q4 2021, and the within-group contribution rate is relatively high in Q3–Q4 2023. However, the overall Theil Index remain low and the equity performance is favorable. Therefore, there is no need for deliberate judgment and intervention on the between-group and within-group disparities.

### 4.2 Safeguarding gig workers: evolution of dispatch equity

**Figure 6:**
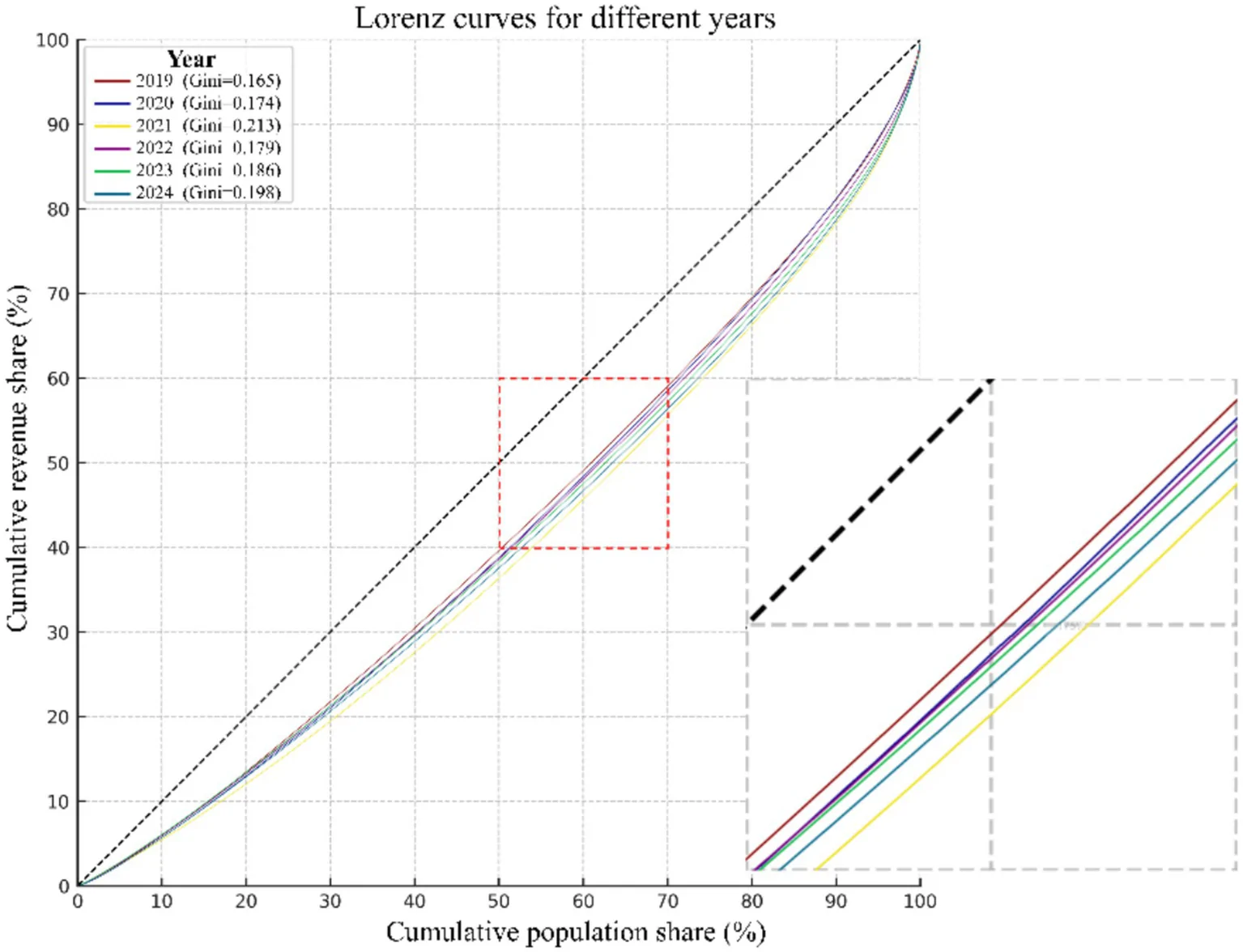
Lorenz Curves of dispatch equity based on drivers’ average revenue per order (2019–2024).

**Figure 7:**
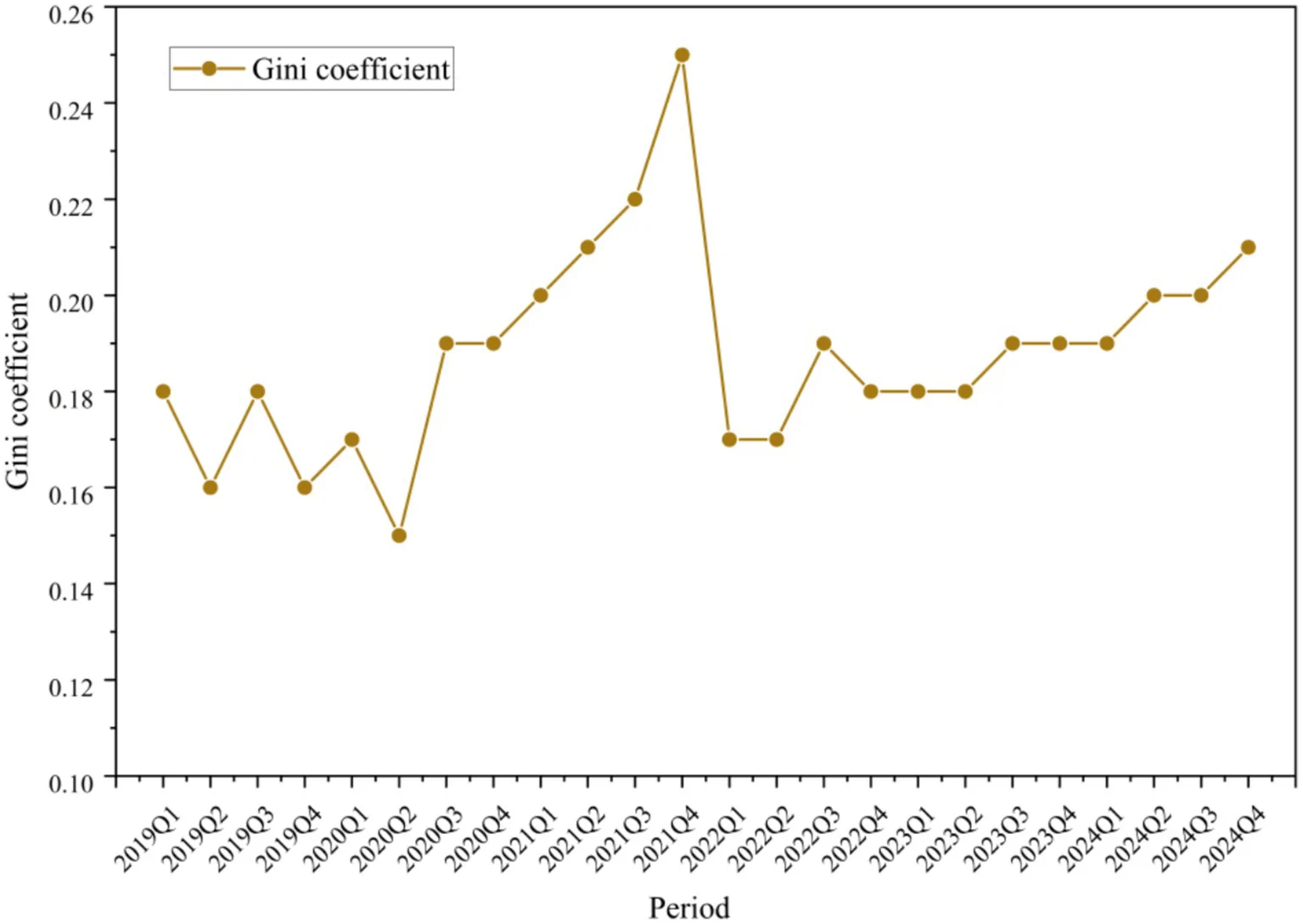
Quarterly evolution of Gini Coefficients for drivers’ average revenue per order (2019–2024).

The livelihoods of ride-hailing drivers—a critical demographic within the gig economy—are highly sensitive to macro-environmental fluctuations. During the observation period (shown in Figures 6, 7), the Gini coefficient for drivers’ average revenue per order fluctuated between 0.165 and 0.213, consistently remaining below the 0.3 threshold.

A longitudinal analysis reveals three distinct phases of equity resilience. Initially (2019 to mid-2020), basic proximity-based dispatch algorithms provided relatively equal opportunities, maintaining a highly equitable environment (Gini dropping to 0.152). However, as the prolonged impacts of the COVID-19 pandemic suppressed overall travel demand, equity resilience sharply deteriorated from late 2020 through 2021, with the Gini coefficient peaking at 0.245. To maintain operational efficiency during the crisis, platforms likely employed incentive-based algorithms prioritizing high-performing drivers, thereby sacrificing income equity for the broader labor pool.

Following government-led cybersecurity reviews and regulatory rectifications starting in Q3 2021, platform algorithms were optimized, leading to a temporary recovery in equity (Gini improving to 0.174). Nevertheless, as the post-pandemic market gradually saturated with an influx of new job-seekers, fierce competition once again pressured platforms to adopt biased dispatching, highlighting the ongoing tension between platform efficiency and driver welfare.

### 4.3 Deconstructing multi-dimensional resilience mechanisms

To further validate the effectiveness of the PSO-PP model in this weighting scenario, this study compares its calculation results with those derived from the entropy weight method (see Table 3). The results indicate that while the two methods yield relatively similar weight distributions for most indicators, significant differences (with an absolute difference greater than 0.05) emerge across three key indicators. This discrepancy profoundly reflects the fundamental distinctions in the core mechanisms of the two methods:

(1) The entropy weight method tends to underestimate core indicators: Taking the “average monthly net profit per vehicle” as an example, this indicator directly dictates the driver retention rate and the stable operation of the industry, serving as a core determinant of sustainable development. However, due to its relatively small fluctuation amplitude across the time series, the entropy weight method assigns it an extremely low weight of 0.0206 (ranking 19th). In contrast, the PSO-PP model accurately identifies its structural importance, assigning it the highest weight (0.1170, ranking 1st).
(2) The entropy weight method tends to overestimate high-volatility indicators: The entropy weight method allocates abnormally high weights to the “total number of leasing companies” and the “average monthly fuel cost per vehicle” (ranking 1st and 3rd, respectively). In reality, the growth in the number of leasing companies often represents blind market expansion and intensified competition, while fuel costs are heavily constrained by external energy prices; neither metric accurately represents the internal operational quality of the industry. By assigning weights solely based on the degree of data dispersion, the entropy weight method disproportionately amplifies the impact of these high-volatility, low-explanatory-power indicators.
(3) The PSO-PP model aligns better with the practical logic of the industry: Conversely, the PSO-PP model assigns higher weights to indicators such as average monthly net profit per vehicle, total quarterly served orders, total number of operational vehicles, average daily revenue per vehicle, and driver dispatch equity. These indicators precisely capture profitability, demand scale, operational efficiency, and service quality, which are highly consistent with the core connotations of sustainable development in the ride-hailing industry.

**Table 3:** Weights of sub-indicators by PSO-PP and entropy weight method separately.

Sub-indicator	Weight coefficient by PSO-PP	Rank	Weight coefficient by EWM	Weight difference
Average monthly net profit per vehicle	0.1170	1	0.0206	0.0964
Total served orders	0.0824	2	0.0631	0.0193
Number of online operational vehicles	0.0734	3	0.0585	0.0149
Average daily revenue per vehicle	0.0717	4	0.0924	0.0207
Driver dispatch equity	0.0668	5	0.022	0.0448
Average passenger waiting time	0.0661	6	0.0395	0.0266
Number of ride-hailing platforms	0.0653	7	0.0724	0.0071
Passenger waiting time equity	0.0538	8	0.0451	0.0087
Average daily passenger-carrying time per vehicle	0.0472	9	0.0527	0.0055
Average monthly vehicle leasing fee	0.0456	10	0.0508	0.0052
Number of driver complaints received by platforms	0.0447	11	0.0317	0.0130
Vehicle utilization	0.0435	12	0.0448	0.0013
Number of ride-hailing leasing companies	0.0416	13	0.0949	0.0533
Number of registered ride-hailing vehicles	0.0390	14	0.0571	0.0181
Daily served orders per vehicle	0.0374	15	0.0551	0.0177
Fuel cost per kilometer	0.0328	16	0.0894	0.0566
Average daily passenger-carrying distance per vehicle	0.0300	17	0.0513	0.0213
Average daily working hours per vehicle	0.0287	18	0.0365	0.0078
Average monthly insurance fee per vehicle	0.0126	19	0.0221	0.0095

Although the entropy weight method offers the advantages of objectivity and computational simplicity, its over-reliance on data dispersion makes it highly susceptible to monotonic time-series trends, local fluctuations, and external disturbances. In comparison, the PSO-PP model is capable of extracting key information that exerts a comprehensive impact by analyzing the holistic structure of multi-dimensional data. Therefore, within the long-term, multi-dimensional evaluation context of this study, adopting the PSO-PP model to determine indicator weights provides superior practical interpretability and methodological applicability.

**Figure 8:**
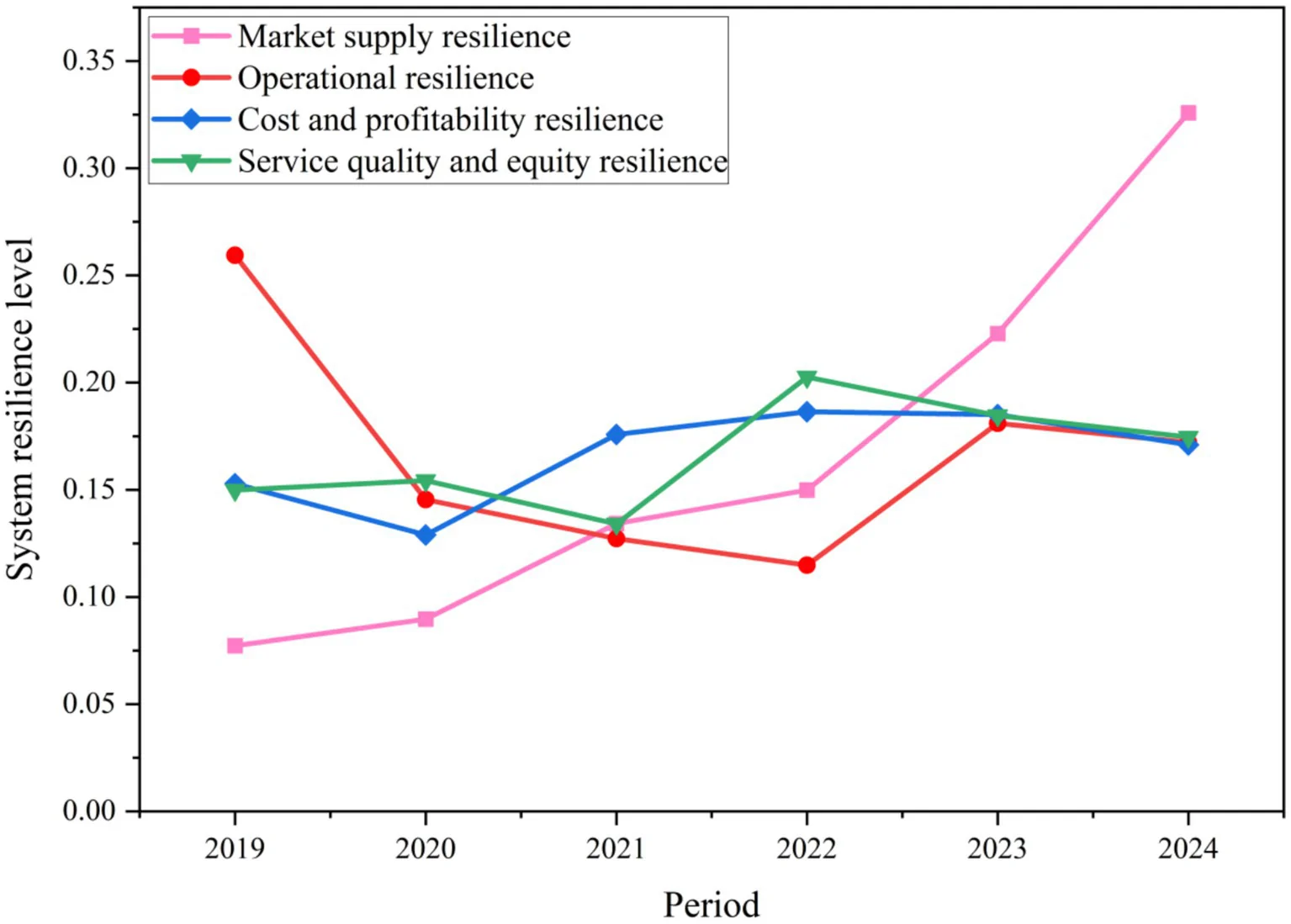
Annual variation trends of four-dimensional resiliencies.

To systematically deconstruct the underlying driving mechanisms of the resilience level of the ride-hailing industry in Ningbo, we conducts a trend analysis of the four primary indicators from 2019 to 2024 (shown in Figure 8).

(1) Market supply resilience.

Market supply resilience climbed from 0.0773 in 2019 to 0.3259 in 2024, driven directly by concurrent expansions in registered vehicles, operating platforms, and leasing companies. The fundamental reason behind this growth is the sector’s inherently low entry barrier. Initially, the rise of aggregation platforms (such as Amap and Meituan) lowered user acquisition costs, and thus contribute to market expansion. This foundational supply surged after 2023, achieving an average annual growth rate of 48.69%. As broader macroeconomic pressures altered the employment landscape (30), the gig economy’s low threshold converted the ride-hailing sector into a primary labor absorption buffer, drawing in massive individuals of job seekers.

(2) Operational resilience.

Operational resilience followed a double-dip trajectory of decline, rebound, and secondary decline between 2019 and 2024. The initial drop from a peak of 0.2594 in 2019 to 0.1148 in 2022 stemmed directly from pandemic-induced demand shocks. Post-pandemic reopening in 2023 stimulated a 67.8% rebound in overall business volume. During this stage, although vehicle-level efficiency lagged significantly, with daily carrying distance and trips increasing by only 16.2 and 8.0%, it remains contributing to the operational resilience rebound. The subsequent decline in 2024 was triggered by a supply–demand mismatch where vehicle surge fundamentally outpaced passenger growth. Because per-vehicle productivity metrics constitute the core sub-indicators of this dimension, their dilution inherently dragged the overarching resilience score downward, locking the sector into structural involution characterized by scale expansion without efficiency gain.

(3) Cost and profitability resilience.

Cost and profitability resilience climbed from 0.1528 in 2019 to 0.1851 in 2023 before experiencing its first absolute decline to 0.1710 in 2024. The multi-year upward trend was driven by dual cost-reduction mechanisms. Rapid fleet electrification, with EV representation surging from 44.8 to 92.4%, systematically compressed fuel expenses per kilometer. Concurrently, upstream price reductions in vehicle manufacturing combined with high-volume leasing strategies successfully lowered monthly driver contract fees. Although these operational buffers absorbed significant financial pressure, their protective capacity was ultimately overwhelmed by a widening supply–demand mismatch. The resulting decline of driver net profit forces the downward turn of this dimension’s resilience in 2024.

(4) Service quality and equity resilience.

Service quality and equity resilience experienced a moderate increase from 0.1499 in 2019 to 0.1746 in 2024. An expanding fleet and more operating platforms steadily reduced passenger waiting times, which supported this overarching growth. A sharp drop occurred in 2021, when both driver dispatch fairness and passenger waiting time equity reached their lowest levels. Strict government supervision in 2022 successfully corrected these imbalances. The resulting push for standardized operations restored market fairness and lifted the overall metric to a new peak.

### 4.4 Overall system resilience trajectory

Based on the closeness values calculated using the TOPSIS evaluation method, the quarterly industry resilience levels and corresponding rankings are shown in Table 4. The resilience development trajectory shows two critical turning points, with the peak value occurring in Q4 2023 (closeness value of 0.620) and the lowest point in Q1 2020 (closeness value of 0.290). The optimal resilience status in Q4 2023 is attributed to strong performance across all resilience dimensions of the ride-hailing industry. Several key indicators performed well. For example, the normalized scores for total served orders, monthly average vehicle leasing fee, average passenger waiting time, fuel cost per kilometer, and passenger waiting time equity were all above 0.8. The closeness value in Q3 2023 ranked second among all quarters, indicating that the second half of 2023 marked a strong period for the industry’s resilience enhancement and stable development.

**Table 4:** Quarterly system resilience level of the ride-hailing industry.

Quarter	C	Rank	Quarter	C	Rank
2019Q1	0.502	15	2022Q1	0.449	21
2019Q2	0.461	19	2022Q2	0.549	10
2019Q3	0.524	14	2022Q3	0.595	3
2019Q4	0.584	7	2022Q4	0.494	16
2020Q1	0.290	24	2023Q1	0.528	12
2020Q2	0.491	17	2023Q2	0.562	9
2020Q3	0.527	13	2023Q3	0.609	2
2020Q4	0.454	20	2023Q4	0.620	1
2021Q1	0.436	22	2024Q1	0.592	4
2021Q2	0.487	18	2024Q2	0.586	6
2021Q3	0.528	11	2024Q3	0.587	5
2021Q4	0.434	23	2024Q4	0.579	8

In contrast, the poorest performance was in Q1 2020, likely due to lockdowns during the early COVID-19 pandemic, a period that severely undermined the comprehensive resilience of the ride-hailing industry. During this period, indicators related to market supply resilience, operational resilience, and service quality and equity resilience all performed poorly. Normalized scores for indicators such as vehicle utilization, total quarterly served orders, monthly net profit per vehicle, monthly vehicle leasing fee, daily average passenger-carrying distance per vehicle, and number of platforms were all below 0.1, reflecting the extreme vulnerability of the industry’s core resilience metrics under large-scale public health emergencies. Additionally, the second-lowest closeness value appeared in Q4 2021, likely due to strict lockdown measures in Ningbo’s Zhenhai District during another wave of the pandemic, which once again posed a severe challenge to the stability of the industry’s resilience level.

Overall, the analysis suggests that external shocks and policy changes have a significant impact on the comprehensive resilience level of the ride-hailing industry.

By 2024, the growth rate of ride-hailing demand began to slow down. However, the number of vehicles entering the industry continued to rise rapidly. This structural imbalance between supply and demand suggests that the market is nearing saturation, which is reflected in the gradual decline of closeness values following their peak in 2023, and further indicates that the industry’s resilience enhancement is facing new bottlenecks under the supply–demand mismatch scenario.

## 5 Discussion and policy implications

The longitudinal analysis of Ningbo’s ride-hailing industry from 2019 to 2024 exposes the profound vulnerability and dynamic adaptive capacity of urban mobility systems under severe public health shocks. To build crisis-resilient cities, it is imperative to move beyond traditional transport management and integrate shared mobility into the broader framework of urban emergency response and public health planning.

(1) Establish a dynamic supply–demand regulation mechanism to safeguard operational resilience.

To build crisis-resilient cities, municipal authorities must treat shared mobility networks as dynamic emergency infrastructure rather than purely hands-off commercial markets. Because unchecked vehicle surge severely degrades systemic efficiency during external macro-shocks (42), transport management must shift toward active supply balancing. Regulators should deploy real-time data monitoring to implement merit-based entry and withdrawal thresholds for platforms, vehicles, and drivers. This ensures the active fleet scales dynamically with actual urban transport demand. Concurrently, local governments should construct an early warning system to detect market saturation and extreme internal competition. Triggering statutory interventions under the Anti-Unfair Competition Law when capacity crosses critical thresholds can halt predatory price wars, prevent systemic operational collapse, and maintain a stable transport buffer for public health emergencies.

(2) Promote intelligent operations and autonomous technologies to reinforce operational adaptability.

To ensure urban mobility networks function reliably during public health emergencies, transport management must incorporate predictive artificial intelligence into municipal emergency planning. Rather than passively reacting to immediate order fluctuations, platforms should deploy advanced spatial–temporal forecasting models to anticipate mobility surges across critical urban sectors (43). Preemptively rebalancing fleets toward healthcare facilities and essential transit hubs directly shortens average passenger waiting times, reduces empty cruising mileage, and maximizes vehicle productivity under severe macro-shocks. Furthermore, long-term public health planning should actively support the strategic integration of autonomous ride-hailing vehicles. Deploying driverless fleets establishes an uncompromised physical transport buffer during infectious disease outbreaks, permanently driving down operational costs while protecting public safety by eliminating human-to-human transmission risks.

(3) Regulate platform take-rates and institutionalize net-profit benchmarks to safeguard the emergency labor reserve.

Our PSO-PP model demonstrates that average monthly net profit serves as the core structural support of overall mobility resilience. To prevent the ongoing erosion of gig earnings from influencing the urban emergency labor pool, transport regulators must shift from managing consumer fares to strictly governing platform extraction. Municipal authorities should enact statutory commission ceilings audited through real-time platform data interfaces. Concurrently, local bureaus should incorporate baseline driver profitability into annual platform licensing evaluations, mandating that operational algorithms maintain median driver net incomes above the regional living wage. For acute public health shocks, governments should establish a localized contingency fund financed jointly by municipal emergency budgets and platform revenue allocations. Using this reserve to directly subsidize active drivers secures a mobilized transport workforce when standard market incentives fail.

(4) Review platform algorithms to ensure fair work and wide service access.

Our study shows that order distribution becomes increasingly unfair to gig drivers during prolonged market downturns. Instead of waiting for platforms to correct themselves, local regulators should actively enforce existing national labor guidelines. Authorities need to inspect platform algorithms to see exactly how ride requests are handed out. Supervision should prevent platforms from using biased dispatch rules that leave new or lower-rated drivers with almost no work during broader crises. Guaranteeing a basic daily minimum of orders for all active drivers keeps this vital workforce from quitting the market. Ultimately, separating order assignment from purely commercial driver ratings ensures that residents in outer urban neighborhoods can still get essential rides during a public health emergency.

## 6 Conclusion

This study quantifies the socio-ecological resilience evolution of the shared mobility sector across four core dimensions: market supply, operational status, cost-profitability, and service equity. By integrating a tensor decomposition approach with a projection pursuit-weighted TOPSIS model, this research longitudinally tracks the system’s trajectory from initial pandemic collapse (Q1 2020) to structural, yet imbalanced, recovery (Q4 2023).

The findings indicate that while the ride-hailing system survived the acute shocks of COVID-19, its long-term resilience is currently threatened by a structural mismatch between stagnating demand and blind supply expansion, alongside persistent spatial–temporal service inequalities. To mitigate these vulnerabilities, strict government interventions have proven effective in restoring market fairness and service equity.

Future studies could apply the evaluation framework and methodologies developed in this research to ride-hailing markets in other cities or regions to test its generalizability. Furthermore, as monitoring technologies advance, future evaluations could include other public transportation modes. These modes could include traditional taxis, subways, regular buses, and shared bicycles. It would help reflect the overall dynamics and internal structural evolution of the urban public transport system, thereby expanding the research scope of resilience assessment from a single ride-hailing industry to the entire urban public transport system.

## Data Availability

The raw data supporting the conclusions of this article will be made available by the authors, without undue reservation.
